# Total adiponectin in overweight and obese subjects and its response to visceral fat loss

**DOI:** 10.1186/s12902-019-0386-z

**Published:** 2019-06-03

**Authors:** Salah Gariballa, Juma Alkaabi, Javed Yasin, Awad Al Essa

**Affiliations:** Department of Internal Medicine, College of Medicine and Health Sciences, United Arab Emirates University, Al Ain, United Arab Emirates

**Keywords:** Adiponectin, Obesity, Visceral fat, Diet

## Abstract

**Background:**

Visceral obesity and related diabetes is reaching epidemic proportions in the United Arab Emirates (UAE). Adiponectin is a hormone that is secreted by adipose tissue and may play an important role in obesity-related morbidity. The aim of this study was to investigate total adiponectin levels in overweight and obese UAE subjects visiting health care facilities for weight management.

**Methods:**

All overweight and obese subjects visiting community health centers were invited to take part in the study. Two hundred and six participants received individualized structured dietary education for weight management. Demographic data, anthropometric measurements and fasting venous blood samples were taken for measurements of total adiponectin and markers of inflammation and nutritional status at baseline and follow up. Multivariate analysis was performed to determine the independent effects of prognostic factors on serum adiponectin levels.

**Results:**

A total of 193 (93%) females with a mean age (±SD) 36 ± 11 years were included in the analysis. During a follow up period of 427 ± 223 days, participants received 13 ± 5 structured dietary education sessions. We observed decreased levels of total adiponectin with increasing quartiles of both waist circumference (WC) and body mass index (BMI). Male gender and history of both gestational and type 2 diabetes were associated with significantly lower total adiponectin levels (*p* < 0.05). After adjusting for age, gender, BMI and hip circumference, multiple regression analysis revealed a significant and independent association between waist circumference and total adiponectin levels. At follow up visceral fat loss was associated with a significant decrease in inflammatory markers and a non-significant increase in total adiponectin levels.

**Conclusion:**

Increased visceral fat in overweight and obese subjects is associated with decreased total adiponectin levels. The health benefits of increasing adiponectin levels using different dietary intervention strategies need to be explored in larger studies.

**Trial registration:**

NCT01691365, registered on 11/09/2012.

## Background

The prevalence of obesity is increasing worldwide and in some Gulf countries such as the United Arab Emirates (UAE), obesity related diabetes is reaching epidemic proportions [1–3]. Recent evidence, including our own work, suggests that visceral obesity in the UAE is more common and closely related to morbidity. Furthermore body mass index (BMI) may underestimate obesity related chronic disease risks [4–7]. A number of inflammatory cytokines secreted by visceral fat have been implicated in obesity-related complications [2]. For example, adiponectin a hormone secreted by the adipose tissue has been found to be low in obese patients and plays an important role in the etiology of type 2 diabetes [8]. Besides its insulin sensitizing effects adiponectin is also known to have anti-inflammatory, antioxidant and cardiovascular modulating effects [9]. Adiponectin an adipocyte-derived hormone may therefore have the potential to provide an important therapeutic tool to reduce the burden associated with obesity and related chronic diseases including diabetes and cardiovascular disease (CVD). Preliminary evidence points to increased adiponectin levels as a result of different dietary intervention strategies and associated weight loss, however, given the paucity of evidence and population differences in adiponectin values further research is still needed to investigate the role of adiponectin in obese subjects with high CVD risk in different ethnic groups [10–13]. The current prevalence of obesity and related diabetes mellitus in our population are among the highest in the world however, still factors that affect obesity and associated morbidities, including diabetes and CVD remain unclear. The objective of this study was to determine adiponectin levels in overweight and obese UAE subjects visiting health care facilities for weight management.

## Methods

All overweight and obese subjects visiting community health centre over a 12-month period for obesity management in Al Ain city, serving a total population of 600,000 were invited to take part in this longitudinal prospective study. Following informed written consent eligible subjects had anthropometric measurements and a fasting 10 ml of blood taken for measurement of adiponectin, markers of inflammation including TNF, IL6, high sensitivity C-reactive protein (hsCRP) and other related nutritional and biochemical variables at baseline. All recruited subjects received structured dietary education to increase their fruit and vegetable consumption and reduce their high-energy food intake by 2 experienced dieticians running separate clinics. Each dietician met individually with every participant for 20 and 30 min per visit to discuss ways of increasing fruit and vegetable consumption and reduce their calorie intake using educational materials to facilitate understanding and adherence. Subjects were managed according to standard practice and followed up once a month for further education lessons.

Inclusion criteria included subjects aged 18 years and over with BMI > 25. Individuals with severe chronic clinical or psychiatric disease, participating in other intervention trials, on dietary supplements or taking anti-obesity medications and those unable to give an informed written consent were excluded. The local research ethical committee has approved the study. **Measurements** Overweight and obesity were diagnosed using WHO non-Asian population sex-adjusted cut-off-points. Baseline clinical assessment including demographic and clinical data was obtained from all subjects. Body weight and height were measured using a Tanita body composition analyzer. Waist circumference (WC) was measured using a flexible plastic tape at the mid-point between lower ribs and iliac crest to the nearest 0.1 cm. ***Blood samples***: Methods for the biological measurements have been published previously [5]. Briefly fasting blood samples were drawn into potassium EDTA vacutainer and one plain tube. Tubes were mixed and centrifuged immediately for 10 min at 4000 rotations/minute. Plasma and serum were collected and stored at − 80 °C. Total adiponectin was measured using the Quantikine immunoassay. This is an enzyme-linked immunosorbent assay (ELISA) test designed to measure total (low, middle and high molecular weight) human adiponectin in serum and plasma (R& D systems, Minneapolis, MN 55413, USA; www.RnDSystems.com). We also used ELISA kits to measure the inflammatory markers TNF and IL6. High sensitivity C-reactive protein (hsCRP) was measured using an Integra 400 Plus automated analyzer (Roche Diagnostics, Mannheim, Germany).

Statistical analyses were performed with SPSS software, version 19.0 (SPSS Inc., Chicago). BMI and WC were divided into 4 equal quartiles. Paired-t test and one-way ANOVA or the nonparametric Kruskal-Wallis H test were used to test within and among-group differences, and *p* value < 0.05 was considered significant. A multivariate analysis was performed to determine the influence of age, gender, BMI, waist and hip circumference on adiponectin levels.

## Results

**Fig. 1:**
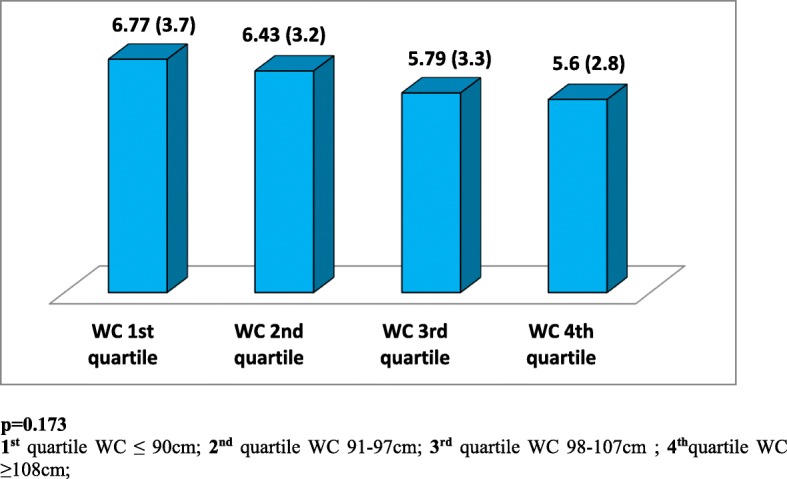
Baseline adiponectin levels (μg/ml), [mean (SD)], according to quartiles of waist circumference (WC) of female subjects

**Fig. 2:**
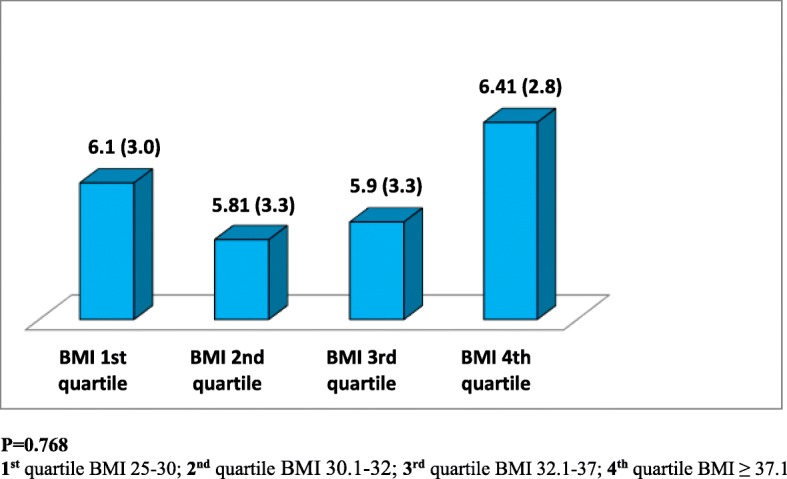
Baseline adiponectin levels (μg/ml), [mean (SD)], according to quartiles of body mass index (BMI) mean (SD) of female subjects

**Fig. 3:**
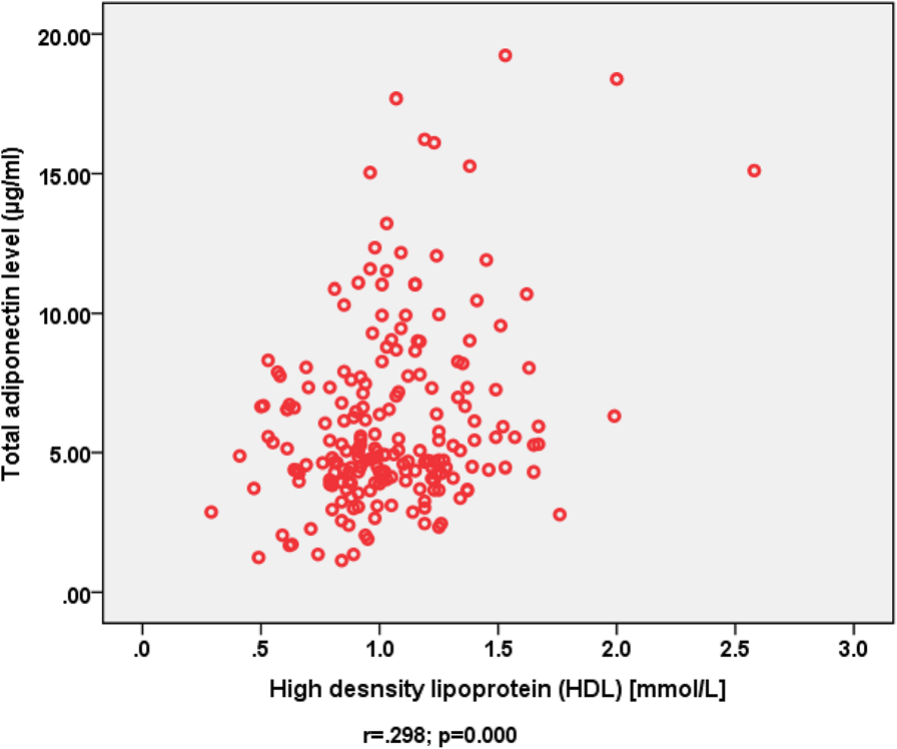
Association between total adiponectin and high density lipoproteins in overweight and obese subjects

A total of 206 overweight or obese subjects (193 female, 13 male) were recruited. Although all 206 subjects had follow up data only 125 subjects agreed to provide follow up blood samples. The study population mean (SD) age was 36 (11) years. Among the 206 subjects recruited 7 (3.4%) had type 2 diabetes, 32 (16%) had gestational diabetes, 10 (5%) had dyslipidemia and 19 were taking medications for diabetes and dyslipidemia. Overweight and obese subjects had mean (SD) dietary education sessions of 13 ± 5 during the follow up period of 427 ± 223 days. Figures 1 and 2 show baseline total adiponectin levels of overweight and obese female subjects according to BMI and WC divided into 4 quartiles. Total adiponectin levels decreased with increasing quartiles for both WC and BMI but the associations were not statistically significant (*p* > 0.05). Male gender and history of both gestational and type 2 diabetes were associated with significantly lower adiponectin levels (*p* < 0.050). Multiple regression analysis revealed significant and independent association between waist circumference and adiponectin levels (*p* < 0.050), [Table 1]. Adiponectin also showed positive correlation with HDL (r = .278, *p* = 0.000) [Fig. 3]. The association between total adiponectin and HDL but not other lipid profile markers remains significant after adjusting for gender, diagnosis of diabetes and use of statins. At follow up decrease in WC and BMI were associated with a significant decrease in inflammatory markers but the increase seen in total adiponectin levels was not statistically significant [Table 2].

**Table 1:** Multiple regression result of age, gender, history of diabetes, BMI, waist and hip circumference on adiponectin concentrations

	Adiponectin concentrations	
	Standardized Regression coefficient(95% C.I)	*P* value
Age (years)	−.038 (−.056 to .034)	0.635
Gender	−.141 (− 3.84 to .050)	0.056
Body mass index	.103 (−.047 to .166)	0.274
Waist circumference	−.256 (−.115 to −.008)	0.024*
Hip circumference	.064 (−.036 to .067)	0.556
Diabetes (yes/no)	−1.145 (−3.45 to .024)	0.053

* *P* value < 0.05

**Table 2:** Baseline and follow up anthropometric, adiponectin and other markers of inflammation at baseline and follow up [Mean (SD)]

Mean (SE)	Baseline (*n* = 125)	Follow up (*n* = 125)	*P* values*
	Baseline	Follow up	
Body mass index	33.5 (5.8)	32.5 (6)	0.000
Waist circumference (cm)	98.9 (13)	95.7 (14)	0.000
Hip circumference (cm)	112.5 (13)	109.5 (14)	0.000
Fat free mass	45.9 (6)	43.8 (6)	0.000
Fat mass	39.7 (5)	38.3 (6)	0.000
Adiponectin (μg/ml)	6.141 (3.4)	6.227 (3.4)	0.838
Hs CRP (mg/l) α	7.1 (7.9)	5.8 (6.5)	0.060
IL6 (pg./ml) α	2.5 (1.72)	2.11 (1.78)	0.0160
TNFα (pg./ml) α	1.41 (0.5)	0.84 (0.4)	0.000

*α* inflammatory markers, *CRP* C-reactive protein (measure of inflammation), *IL6* Interleukin 6

## Discussion

Our results suggest an inverse association between visceral fat and total adiponectin levels. We also found visceral fat loss was associated with a significant decrease in inflammatory markers and a non-significant increase in total adiponectin levels at follow up. These results add to our previously reported findings of increased inflammation and decreased antioxidant status in UAE subjects with visceral obesity [4, 5].

Given that abdominal obesity is more common in the UAE population and that the UAE has one of the highest prevalence of obesity related diabetes mellitus in the World these new preliminary findings may have important public health implications [1, 3]. This is because a number of factors secreted by visceral fat are implicated in the pathologies associated with obesity [2, 5–7]. For example, adiponectin a hormone one of many adipokines secreted by adipose tissue with a number of endocrine and paracrine effects on the cardiovascular system [9]. Adiponectin has insulin sensitizing properties and is thought to improve insulin sensitivity, cellular glucose uptake and reduce hepatic gluconeogenesis [8]. In addition adiponectin has been reported to have anti-inflammatory effects and reducing cardiovascular oxidative stress [9]. The role of adiponectin in insulin resistance and cardiovascular homeostasis is still not fully understood. Furthermore, the beneficial cardiovascular effects of high circulating adiponectin do not appear to translate into better clinical outcomes in patients with CVD and further research is therefore warranted [8]. Nevertheless the anti-inflammatory and anti-diabetic protective effects make adiponectin a promising therapeutic target [8, 9].

The relationship between adiponectin and insulin sensitivity is influenced by ethnic differences and was found to vary between different populations [10, 14]. In a south Asian population for example, which share some similarities with our study population, adiponectin was found to be associated with adiposity and insulin resistance [15]. Regional and ethnic differences in adiponectin values and other related adipokines may have an important role to play in the susceptibility of certain populations to obesity and associated CVD morbidities [11]. Because available research data in this area is limited more research involving different populations is needed.

We found both gestational (GDM) and type 2 diabetes in our study population were associated with lower adiponectin levels compared with healthy controls [16, 17]. A recent meta-analysis of prospective studies assessed the association of levels of adiponectin and other inflammatory markers with risk of type 2 diabetes, reported strong association between elevated inflammatory markers, low levels of adiponectin and risk of type 2 diabetes. However, the authors reported considerable heterogeneity between studies and suggested that more research is required to clarify the relationship between adipokines, including adiponectin and the risk of type 2 diabetes as well as its therapeutic potentials [17].

The positive association between adiponectin levels and HDL is consistent with previous studies [18, 19]. Given the known association between low levels of HDL and increased risk of CVD and reduced protective effects of HDL in patients with type 2 diabetes this finding may be of clinical interest [14]. The lack of association between adiponectin and other lipid profile markers may partly be explained by the use of statins known to reduce LDL-cholesterol and less likely to affect HDL levels.

The small increase in total adiponectin levels coupled with decreased WC (visceral fat); as a result of dietary education did not reach statistical significance. The average decrease in WC achieved in our study population was around 3%. Although traditionally recommended weight loss target to improve health is around 5%, recent evidence suggests that a fall in body weight as small as 1 kg can improve health if this achieves permanent change in the person’s weight trajectory [20, 21]. More importantly, some preliminary observations suggest that nutrition interventions such as calorie-restriction, Mediterranean diet and garlic extract administration may increase adiponectin concentrations [11]. A preliminary feasibility study in 32 normal and overweight subjects randomized to 12-week alternate day fasting or a control group eating ad libitum revealed reduced total body weight and fat mass coupled with increased plasma adiponectin in the intervention group compared with the control group [12]. In another study weight and visceral fat mass reduction in severely obese patients following bariatric surgery resulted in increased serum adiponectin levels with no significant correlations between changes in adiponectin and body mass index and visceral fat measured using waist circumference [13]. A recent long-term weight reduction trial, using 4 different dietary intervention approaches reported increased adiponectin levels and improved fat distribution and lipid metabolism independent of weight change [22]. This study highlights the potential benefits of manipulating adiponectin levels through dietary modifications in obese patients.

More research is needed to elucidate the mechanisms and potential pathways for increasing adiponectin in different populations and more importantly its potential benefit in the treatment and prevention of obesity associated metabolic diseases such as type 2 diabetes .

An important limitation of the study was the reduced numbers of subjects at follow up visits. This was because some subjects refused to provide blood samples. Another limitation was the lack of a control group and the open design. Nevertheless, body composition measurements were performed digitally and printed on a sheet to minimize observer error. Biochemical analyses were also carried out by a laboratory technician not involved in the recruitment, dietary education or outcome data collection.

## Conclusions

In summary our results show that increased visceral fat in overweight and obese subjects is associated with decreased total adiponectin levels. Given that increased visceral fat is also associated with increased oxidative stress and increased inflammatory markers factors such as antioxidant rich diets that favor visceral fat loss could provide an important tool to reduce the burden associated with obesity and related chronic diseases.

## Data Availability

Data is available upon request to the corresponding author.

## Abbreviations

BMI: Body mass index
CVD: Cardiovascular disease
HC: Hip circumference
HDL: High density lipoprotein
Hs CRP: High sensitivity C reactive protein
IL6: Interleukin 6
LDL: Low density lipoprotein
UAE: United Arab Emirates
WC: Waist circumference
